# Hormone- and HER2-receptor assessment in 33,046 breast cancer patients: a nationwide comparison of positivity rates between pathology laboratories in the Netherlands

**DOI:** 10.1007/s10549-019-05180-5

**Published:** 2019-03-01

**Authors:** Carmen van Dooijeweert, Ivette A. G. Deckers, Inge O. Baas, Elsken van der Wall, Paul J. van Diest

**Affiliations:** 1 Department of Pathology, University Medical Center Utrecht, PO Box 85500, 3508 GA Utrecht, The Netherlands; 2 Foundation PALGA (the nationwide network and registry of histo- and cytopathology in the Netherlands), Houten, The Netherlands; 3 Department of Medical Oncology, University Medical Center, Utrecht, The Netherlands

**Keywords:** Invasive breast cancer, Pathology, Hormone receptor, HER2, Inter-laboratory variation, PALGA

## Abstract

**Purpose:**

Patient management of invasive breast cancer (IBC) is to a large extent based on hormone- and HER2-receptor assessment. High-quality, reliable receptor assessment is of key importance as false results may lead to under- or overtreatment of patients. Surveillance of case-mix adjusted positivity rates has been suggested as a tool to identify laboratories with insufficient testing assays, as this covers the whole process of receptor assessment and enables laboratories to benchmark their positivity rates against other laboratories. We studied laboratory-specific variation in hormone- and HER2 positivity rates of 33,046 breast cancer patients using real-life nationwide data.

**Methods:**

All synoptic pathology reports of IBC resection-specimens, obtained between 2013 and 2016, were retrieved from the nationwide Dutch pathology registry (PALGA). Absolute and case-mix adjusted receptor positivity rates were compared to the mean national proportion and presented in funnel plots in separate analyses for estrogen (ER), progesterone (PR) and HER2. Case-mix adjustment was performed by multivariable logistic regression.

**Results:**

33,794 IBC lesions from 33,046 patients of 39 pathology laboratories were included. After case-mix adjustment, mean positivity rates were 87.2% for ER (range 80.4–94.3), 71.3% for PR (62.5–77.5%), and 9.9% for HER2 (5.5–12.7%). Overall, 14 (35.9%), 17 (43.6%) and 11 (28.2%) laboratories showed positivity rates outside the 95% confidence interval for ER, PR and HER2, respectively.

**Conclusion:**

This nationwide study shows that absolute variation in hormone- and HER2-receptor positivity rates between Dutch pathology laboratories is limited. Yet, the considerable number of outlying laboratories shows that there is still need for improvement. Continuous monitoring and benchmarking of positivity rates may help to realize this.

## Introduction

Patient management of invasive breast cancer (IBC) is to a large extent based on estrogen-(ER), progesterone-(PR) and HER2-receptor assessment as they determine whether targeted anti-hormonal, anti-HER2 therapy and/or chemotherapy are indicated [1–4]. For early ER- and/or PR-positive breast cancer, the risk of recurrence and mortality is reduced by anti-endocrine therapy, independent of the administration of chemotherapy [5, 6]. In addition, for HER2-positive breast cancer, adjuvant anti-HER2-therapy combined with chemotherapy is considered, regardless of other characteristics like tumor grade [1]. Furthermore, different chemotherapy regimens are considered for HER2-positive breast cancer patients [1].

ER-, PR- or HER2-receptor status of a tumor is established by pathological analysis of tumor tissue by immunohistochemistry (IHC) (ER, PR and HER2) and/or in situ hybridization (ISH) (HER2) [1, 7], which, according to global guidelines, is mandatory for all newly diagnosed primary IBC cases [1, 2, 4, 8–10]. High-quality, reliable receptor assessment is of key importance as false-negative results may result in withholding effective treatment, whilst false-positive results could result in overtreatment with costly and ineffective therapy at the same time resulting in unwanted direct and long-term side effects [1, 11–16].

The quality of ER-, PR-, and HER2-testing has been extensively studied over the past two decades. Central review of trial cases or cases from local pathology laboratories mainly showed that substantial differences between testing laboratories occurred [3, 17–24], which was confirmed by reversed studies in which samples or tissue microarrays were sent to different laboratories [25–30]. Proficiency testing programs were launched as a promising remedy [31–35], but it has been argued that they render only a temporary and incomplete assessment of testing performance, which does not necessarily reflect reliability of testing over time [7]. For example, crucial steps like tissue fixation and processing are not covered by these tests [36].

Recently, surveillance of positivity rates has been suggested as a tool to identify laboratories with insufficient testing assays and a high yield of false-positive or false-negative results [7, 14, 16, 37]. However, as test accuracy is not the only potential factor in receptor positivity rates, it is important to also take patient and tumor characteristics into account [14, 38]. Such a study design would enable laboratories and pathologists to compare their receptor positivity rates with other laboratories, while controlling for differences in population characteristics (“case-mix”) [38]. This may be crucial to create awareness, as pathologists and their laboratories may feel addressed by their own case-mix adjusted “mirror” data. Previous studies using such a design found significant variation between pathology laboratories in Germany with a range of HER2 positivity rates varying from 7.6 to 31.6% [7, 14] with significant outliers even after case-mix correction [14]. To the best of our knowledge, such studies have not been performed for ER- and PR-receptor positivity rates.

To create insight and awareness in the Netherlands, we compared ER-, PR- and HER2-receptor positivity rates from daily clinical practice between pathology laboratories using real-life data from synoptic (structured) pathology reports of 33,046 IBC patients from the Dutch nationwide pathology registry (PALGA).

## Methods

### Data source and study population

We extracted data from PALGA, the nationwide network and registry of histo- and cytopathology in the Netherlands, which contains pathology reports from all Dutch pathology laboratories since 1991 [39]. Data from the PALGA database are pseudonymized by a trusted third party (ZorgTTP, Houten, the Netherlands). As all pathology laboratories were initially anonymized, we obtained further written consent for the additional analysis of inter-pathologist variation within individual laboratories (*n* = 7). This study was approved by the scientific and privacy committee of PALGA and all data were retrieved and handled in compliance with the General Data Protection Regulation act.

**Fig. 1:**
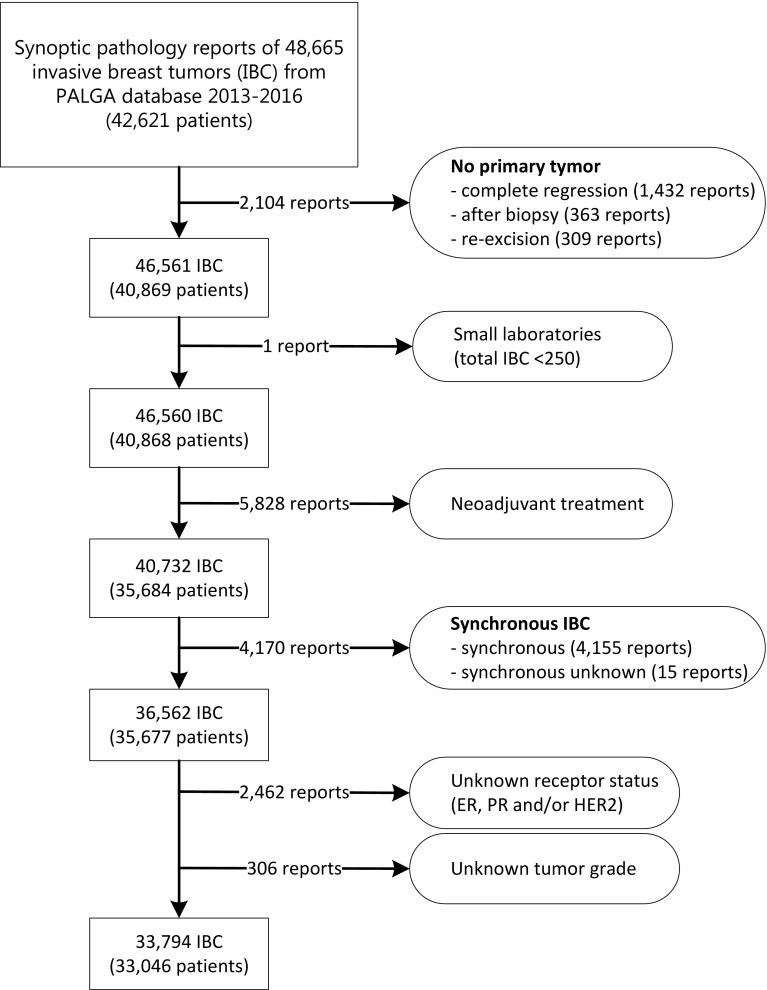
Flowchart of included lesions of invasive breast cancer (IBC) to assess variation in receptor (ER, PR, HER2) positivity rates between laboratories

All synoptic pathology reports of patients with IBC resection specimens between January 1, 2013, and December 31, 2016, in the Netherlands (*n* = 48,665) were extracted. Synchronous IBC was defined as an ipsilateral lesion within six months of the previous IBC resection during the study period. As these lesions were considered paired measurements, we only included the first lesion. Reports of resection specimens without a primary tumor were excluded. Likewise, pathology reports of patients who received neoadjuvant treatment were excluded as tumor receptor status may be converted by neo-adjuvant treatment [40–42] (Fig. 1).

Overall, 40 out of 46 Dutch pathology laboratories reported on breast resection specimens using the synoptic (PALGA) pathology protocol. Of these laboratories, we only included those that synoptically reported ≥ 250 IBC resection specimens during the study period (*n* = 39). For inter-pathologist variation within individual laboratories, we only analyzed data from pathologists from the consenting laboratories who synoptically reported ≥ 20 IBC during the study period.

From each pathology report, we extracted patient characteristics (sex, age, type of surgery) and tumor characteristics (tumor size, histologic subtype, histologic grade, ER- and PR-receptor status, and HER2-receptor status). ER- and PR-status were determined by IHC, whereas HER2-status was determined either by IHC and/or ISH. Lastly, reports of IBC with any missing data (histologic grade, ER, PR- or HER2-receptor status) were excluded from further data-analysis (Fig. 1).

### Analysis of ER- and PR-receptor status

Within the synoptic protocol, and according to the Dutch guideline [1], the ER- and PR-receptor status are considered positive when ≥ 10% of tumor cells show ER- and PR-specific staining by IHC. Overall, both ER- and PR-receptor status were taken into account as a binary variable, either positive (≥ 10%) or negative (< 10%), since the percentage of stained tumor nuclei (not an obligatory item) was not known for ~ 20% of cases. When one or both receptors were missing, the status on biopsy was considered the true receptor status (~ 7.5%), as this is common practice in clinical management.

### Surrogate intrinsic subtype

Surrogate intrinsic subtype was established as before by Perou et al. [43] as Luminal A = ER+, PR+/−, HER2−, Luminal B = ER+, PR+/−, HER 2+, HER2-driven = ER−, PR−, HER2+, Basal-like = ER−, PR−, HER2−.

### Analysis of HER2-receptor status

HER2-receptor status was taken into account as a binary variable, either positive or negative, regardless of which techniques were used (IHC and/or ISH). In general, and as recommended by the Dutch guideline [1], IHC is performed first, followed by amplification testing in case of a 2 + IHC score. As described for ER− and PR, when HER2-receptor status was missing on resection specimen, biopsy HER2-receptor status was considered the true receptor status (5.5%).

### Survey among laboratories

A survey was sent to all 46 Dutch pathology laboratories to gain insight into their processes and interpretation of receptor assessment in daily clinical practice. The survey included questions on whether receptor status was assessed on biopsy and/or resection specimen, the cutoff percentages used for receptor positivity (ER/PR), the interpretation of IHC scores for HER2 (0, 1+, 2+, 3+), techniques used for HER2-assessment and the order in which they were executed.

### Statistical analysis

Separate analyses were performed for ER-, PR- and HER2-receptor status as outcome measure. Patient and tumor characteristics were summarized and differences between receptor-positive and receptor-negative status (ER, PR and HER2) were tested by means of χ^2^ test for categorical variables and by a nonparametric Kruskal–Wallis test for continuous variables.

Overall positivity rates per receptor (ER, PR, HER2) were determined and considered the national proportion. Absolute differences in positivity rates between laboratories were presented in funnel plots per receptor, in which the positive-receptor proportions per laboratory were plotted against the number of included IBC reports per laboratory, with the overall national proportion with its 95% confidence limits as target [44].

For case-mix correction, all available clinicopathological risk factors were selected a priori based on literature [14, 38, 45–47] and on pathologists’ experience. These factors included age, sex, tumor size, type of surgery, histologic subtype, tumor grade and either the combined hormone-receptor status (for HER2-analysis) or HER2-receptor status (for ER- and PR-analysis). The combined hormone-receptor status (ER/PR) was considered positive when either or both the ER- and PR-receptor were reported as positive. Sex was excluded in the final multivariable logistic regression model, as the number of males was too low. However, males did not cluster in specific laboratories. To calculate case-mix adjusted percentages, the observed percentage (O) per laboratory was divided by the expected percentage (E), based on the multivariate logistic regression model, and multiplied by the overall mean positive percentage per receptor (O/E * mean). Similar to the crude percentages, case-mix adjusted percentages were presented in funnel plots.

For analysis of the inter-pathologist variation within the laboratories, we merely compared the proportions per receptor (ER, PR and HER2) between pathologists by Fisher exact test (Monte Carlo option).

Survey results were summarized by frequencies and percentages. *P* values below 0.05 were considered statistically significant. All statistical analyses were performed using IBM SPSS Statistics version 25.

## Results

### Characteristics of patients, DCIS lesions and laboratories

In total, 33,794 unique IBC lesions of 33,046 patients from 39 laboratories were included. Characteristics of all included patients and corresponding invasive breast tumors are listed in Table 1.

**Table 1:** Characteristics of the 33,794 included invasive breast cancers from the Dutch national PALGA database 2013–2016

	Total (*n* = 33,794)	ER-negative (*n* = 4337)	ER-positive (*n* = 29,457)	PR-negative (*n* = 9698)	PR-positive (*n* = 24,069)	HER2-negative (*n* = 30,454)	HER2-positive (*n* = 3340)
Age (year)*	62.2 (12.1)	60.9 (14.2)	62.4 (11.7)	62.9 (12.5)	61.9 (11.9)	62.5 (11.9)	59.6 (13.1)
Sex, *n* (%)							
Female	33,540 (99.2%)	4335 (100.0%)	29,205 (99.1%)	9666 (99.7%)	23,874 (99.1%)	30,219 (99.2%)	3321 (99.4%)
Male	254 (0.8%)	2 (0.0%)	252 (0.9%)	32 (0.3%)	222 (0.9%)	235 (0.8%)	19 (0.6%)
Tumor size (cm)*	1.9 (1.3)	2.2 (1.6)	1.8 (1.3)	2.0 (1.5)	1.8 (1.3)	1.8 (1.3)	2.1 (1.4)
Type of surgery, *n* (%)							
Mastectomy	12,208 (36,1%)	1902 (43.9%)	10,306 (35.0%)	3961 (40.8%)	8247 (34.2%)	10,641 (34.9%)	1567 (46.9%)
Breast conserving	21,586 (63.9%)	2435 (56.1%)	19,151 (65.0%)	5737 (59.2%)	15,849 (65.8%)	19,813 (65.1%)	1773 (53.1%)
Histologic subtype, n (%)							
Ductal	28,549 (84.5%)	3763 (86.8%)	24,786 (84.1%)	8137 (83.9%)	20,412 (84.7%)	25,416 (83.5%)	3133 (93.8%)
Lobular	4429 (13.1%)	95 (2.1%)	4334 (14.7%)	1,012 (10.4%)	3417 (14.2%)	4291 (14.1%)	138 (4.1%)
Other	816 (2.4%)	479 (11.0%)	337 (1.1%)	549 (5.7%)	267 (1.1%)	747 (2.5%)	69 (2.1%)
Histologic grade, *n* (%)							
Grade 1	9494 (28.1%)	130 (3.0%)	9364 (31.8%)	1487 (15.3%)	8007 (33.2%)	9283 (30.5%)	211 (6.3%)
Grade 2	16,103 (47.1%)	964 (22.2%)	15,139 (51.4%)	3696 (38.1%)	12,407 (51.5%)	14,767 (48.5%)	1336 (40.0%)
Grade 3	8197 (24.3%)	3243 (74.8%)	4954 (16.8%)	4515 (46.6%)	3682 (15.3%)	6404 (21.0%)	1793 (53.7%)
Combined ER/PR status, *n* (%)							
Negative	4216 (12.5%)	–	–	–	–	3179 (10.4%)	1037 (31.0%)
Positive	29,578 (87.5%)	–	–	–	–	27,275 (89.6%)	2303 (69.0%)
HER2-receptor status, *n* (%)							
Negative	30,454 (90.1%)	3265 (75.3%)	27,189 (92.3%)	7,866 (81.1%)	22,588 (93.7%)	–	–
Positive	3340 (9.9%)	1072 (24.7%)	2268 (7.7%)	1832 (18.9%)	1508 (6.3%)	–	–
Intrinsic subtypes**							
Luminal A	27,189 (80.5%)	–	–	–	–	–	–
Luminal B	2.268 (6.7%)	–	–	–	–	–	–
HER2-driven	1.072 (3.2%)	–	–	–	–	–	–
Basal-like	3.265 (9.7%)	–	–	–	–	–	–

* Mean (SD)

** Luminal A = ER+, PR+/–, HER2–, Luminal B = ER+, PR+/–, HER 2+, HER2-driven = ER– , PR–, HER2+, Basal-like = ER–, PR–, HER2–

***P values for all variables (positive versus negative receptor status) < 0.0005, except for males and HER2 (*P* = 0.198)

Nearly all patients were female (99.2%), and the overall mean (± standard deviation (SD)) age was 62.2 (± 12.1) years. The majority of patients underwent breast conserving surgery (63.9%) for tumors with a mean (± SD) of 1.9 (± 1.3) cm. HER2 positivity of tumors was associated with higher histologic tumor grade, whereas ER- and PR positivity of tumors was associated with lower tumor grade. HER2-positive tumors were less often of lobular subtype and were of larger size than HER2-negative tumors. Furthermore, HER2-positive tumors were less often hormone-receptor positive and vice versa.

The number of synoptically reported IBC lesions per laboratory ranged from 80 to 2224 (median 794). Overall observed positive proportions were 87.2% for ER, 71.3% for PR and only 9.9% for HER2. Regarding the intrinsic breast cancer subtypes, luminal A, luminal B, HER2-driven and basal-like subtypes were observed in 80.5%, 6.7%, 3.2% and 9.7%, respectively (Table 1).

### Inter-laboratory variation in ER, PR and HER2 positivity rates

**Fig. 2:**
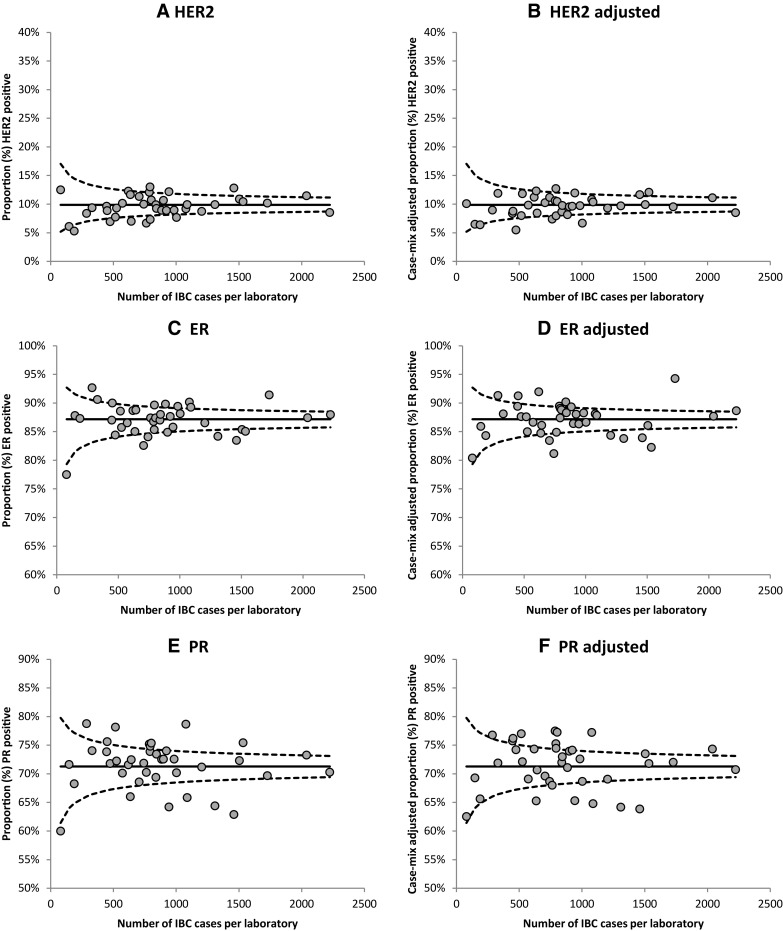
Funnel plots showing the observed (**a, c, e**) and case-mix adjusted positivity rates (**b, d, f**) per laboratory (dots) relative to the mean national proportion and its 95% confidence intervals for, for HER2 (**a, b**), estrogen (ER) (**c, d**) and progesterone (PR) (**e, f**) (2013–2016)

Positivity rates between laboratories varied most for PR (60.0–78.8%), followed by ER (77.5–92.7%) and HER2 (5.3–13.0%). After case-mix adjustment, the inter-laboratory range slightly decreased for all receptors: PR (62.5–77.5%), ER (80.4–94.3%), HER2 (5.5–12.7%) (Fig. 2). Overall, 17 laboratories (43.6%) showed positivity rates outside the 95% CI for PR, followed by 14 laboratories (35.9%) for ER and 11 laboratories (28.2%) for HER2 (Fig. 2).

### Intra-laboratory variation in ER, PR and HER2 positivity rates

Sixty-two pathologists from the seven laboratories that participated in the intra-laboratory variation analysis synoptically reported ≥ 20 IBC during the study period. Per laboratory the number of analyzed pathologists ranged from 3 to 15 (median 9). The number of analyzed IBC reports per pathologist ranged from 20 to 257 (median 81). Overall, positivity ranges for ER, PR and HER2 did not significantly differ between pathologists within individual laboratories, except for ER positivity rates of the three pathologists from one laboratory (laboratory 10, positivity rates 90.1/98.8/92.9%, *P* = 0.032, data not shown).

### Results of survey

Thirteen of the 46 Dutch pathology laboratories (28.3%) responded to our online survey, of which six were academic laboratories (Table 2). All responding laboratories participated in mandatory external audits (SKML, NordiQC and/or UK-Neqas) and all IHC stainings were executed mechanically. The vast majority of responding laboratories (92.3%) currently performs receptor assessment on biopsy, which is usually only repeated on resection specimen in case of a negative staining. In accordance with the Dutch national guideline (1), all laboratories, except for one, use 10% as a cutoff for ER and PR positivity. The laboratory that uses a different cutoff percentage, i.e., 1%, was not included in our dataset, as they did not synoptically report on breast cancer during the study period. For HER2 testing, 23.1% of the responding laboratories uses an amplification test (FISH), possibly followed by IHC, as primary test. In addition, one academic laboratory performed amplification testing after any plus-score (i.e., 1+, 2+, 3+).

**Table 2:** Responses of 13 laboratories to our survey on receptor assessment of invasive breast cancer

	*n* (%)		
	Total (*n* = 13) (%)	Academic (*n* = 6) (%)	Non-academic (*n* = 7) (%)
Testing on biopsy or resection specimen?			
Biopsy^a^	12 (92.3)	6 (100.0)	6 (85.7)
Resection specimen^b^	1 (7.7)	0 (0.0)	1 (14.3)
Both	3 (23.1)	3 (50.0)	0 (0.0)
Used cutoff for ER- and PR-receptor positivity? (%)			
≥ 1	1 (7.7)	1 (16.7)	0 (0.0)
≥ 10	9 (69.2)	4 (66.7)	5 (71.4)
≥ 11	3 (23.1)	1 (16.7)	2 (28.6)
Used techniques for HER2-receptor assessment^c^			
Immunohistochemistry (IHC)	13 (100.0)	6 (100.0)	7 (100.0)
Fluorescence in situ hybridization (FISH)	7 (53.8)	4 (66.7)	3 (42.9)
Silver in situ hybridization (SISH)	5 (38.5)	3 (50.0)	2 (28.6)
Chromogenic in situ hybridization (CISH)	1 (7.7)	0 (0.0)	1 (14.3)
Multiplex ligation-dependent probe amplification	1 (7.7)	1 (16.7)	0 (0.0)
Next generation sequencing	1 (7.7)	1 (16.7)	0 (0.0)
HER2-receptor: order of testing techniques			
IHC followed by amplification testing	10 (76.9)	4 (66.7)	6 (85.7)
FISH followed by IHC when indicated	3 (23.1)	2 (33.3)	1 (14.3)
Primary IHC HER2-test (*n* = 10) scores			
Score 0			
Reported as negative	10 (100.0)	4 (100.0)	6 (100.0)
Score 1+			
Reported as negative	9 (90.0)	4 (100.0)	6 (100.0)
Additional amplification test	1 (10.0)	1 (25.0)	0 (0.0)
Score 2+			
Additional amplification test	10 (100.0)	4 (100.0)	6 (100.0)
Score 3+			
Reported as positive	8 (80.0)	3 (75.0)	5 (83.3)
Additional amplification test	2 (20.0)	1 (25.0)	1 (16.7)

^a^Most laboratories repeat receptor assessment on resection specimen in case of a negative receptor status on biopsy

^b^Receptor assessment on biopsy only when requested by clinician

^c^≥ 1 answer possible

## Discussion

We studied inter-laboratory variation in ER, PR, and HER2 positivity rates in a nationwide cohort of 33,046 invasive breast cancer patients, using real-life data from synoptic pathology reports of the Dutch nationwide pathology registry (PALGA). The results of this study show that absolute differences of ER, PR and HER2 positivity rates between laboratories were reassuringly limited. However, the number of outlying laboratories after case-mix adjustment for ER (14/39), PR (17/39) and HER2 (11/39) clearly shows that there is still room for improvement.

Overall positivity rates were 87.2% for ER, 71.3% for PR and 9.9% for HER2, which, for ER and PR, is in line with previous studies [48–50], whereas for HER2 this is somewhat lower than the percentages of 15%–25% that are often referred to [7, 14, 16, 26, 51–53]. Although we only included synoptic pathology reports, there is no reason to assume that our synoptic dataset may have been selective, since data from the Dutch Breast Cancer Audit (NBCA), which also holds data from narrative pathology reports, show similar receptor positivity rates [49]. Moreover, over 80% of (pre)malignant breast lesions are currently reported via the synoptic PALGA protocol by Dutch pathologists [54], which results in an increased overall completeness of reports [55] and it enables easy and error-free data extraction. This study stresses the potential of using a population-based registry as it provides information on the actual situation in daily clinical practice, which may differ from data derived from clinical trials, from smaller cohorts or even from neighboring countries.

It could be argued that positivity rates in this study may have been biased for several reasons. First, in case of a missing receptor status on resection specimen, the receptor status of the biopsy, when known from the resection pathology report, was included in the analysis. As, however, discrepancies of receptor status between biopsy and resection specimen are uncommon and, according to literature, results from the core biopsy can be used with confidence [56–60], there is no reason to assume that this has influenced our positivity rates. Secondly, we excluded pathology reports with a missing ER-, PR- or HER2-receptor status (*n* = 2462). For the majority of these missing values (~ 70–75%), the tumor receptor status was reported as “*in progress”*. As IHC staining usually takes overnight, the definitive receptor status may have been added as a narrative addendum to the pathology report afterward, yet not to the synoptic PALGA protocol, and therefore, it is unknown in this dataset. However, it is unlikely that this happens more often to receptor-positive than to receptor-negative tumors. For the remaining 735 reports with missing values, the reason remained unknown.

Overall, receptor positivity rates of individual laboratories were compared to the mean national positivity rates, with and without correction for case-mix. Case-mix adjustment only slightly narrowed the range of positivity rates between laboratories, which indicates that there is either little variation in case-mix per laboratory in the Netherlands, or there is little effect of the included case-mix variables. Either way, case-mix does not explain the inter-laboratory differences in this study. In addition, as laboratories with both few and many reports showed positivity rates outside the 95% CI (Fig. 2), laboratory sample size also does not explain the inter-laboratory variation that was found in this study. Furthermore, variation between individual pathologists within laboratories was minimal, which suggests that factors other than pathologists’ interpretation of the fixed and immunohistochemically stained tissue slides may explain the inter-laboratory differences in receptor positivity rates. One could for example think of different ways of tissue fixation or the use of different antibodies between laboratories.

Despite the low response rate of our survey (13/46 laboratories), it did show that, in spite of a clear national guideline, one of the 13 responding laboratories uses a different positivity threshold for ER and PR, which is undesirable as this would result in different therapy advice in our country, even if two laboratories would estimate the same percentage of ER or PR stained nuclei. As all laboratories in this study are anonymous, the results of our survey could not be linked to the laboratories in the dataset. Therefore, it remains unknown whether the use of different positivity thresholds (1% vs. 10%) may (partially) cause the found inter-laboratory variation. However, we do know that only a fairly small proportion of patients shows “arguable” staining percentages between 1 and 10%. For both ER and PR, the percentage of staining was known in approximately 80% of reports and of those reports, 1.3% showed ER percentages between 1 and 10%, whereas this was the case for 7.5% for PR. Therefore, the overall influence of (possible) different cutoff percentages is probably be limited.

A nationwide multidisciplinary breast cancer audit (NBCA) has already been implemented in breast cancer care in the Netherlands [49], yet currently there is only one pathology indicator, i.e., whether the PALGA protocol is used for reporting on (pre)malignant breast lesions [61]. We believe that it is important to use this synoptic PALGA protocol to monitor and benchmark the major pathology breast cancer biomarkers, namely ER, PR, HER2 and histologic grade, as these are crucial in decision making in current clinical practice [1]. Although molecular or genetic measures of prognosis may become increasingly important in IBC risk stratification in the near future, the only three mandatory breast cancer biomarkers are still ER-, PR- and HER2-receptor status, despite the massive investment of time and money into development of new biomarkers [4]. What is more, Groenendijk et al. [62] showed that the distribution of genomic risk is mainly influenced by histologic grade and ER- and HER2-status, which shows that these classic biomarkers remain very relevant. Given their prominent role in clinical practice, it seems worthwhile to invest in better and more uniform assessment of these classic biomarkers.

We believe that creating insight and awareness in variation of clinically relevant biomarkers through annual individual pathology “mirror” reports is an important step toward improvement in breast cancer care. Monitoring the receptor positivity rates may help to identify laboratories with a high number of false-positive or false-negative results [7, 14, 16, 37, 38] that are not picked up by the external audits, since crucial steps like tissue fixation and processing are not covered by these tests [36]. Furthermore, pathologists and their laboratories may feel best addressed by their own, case-mix adjusted, “mirror” data visualized against other national laboratories. Indeed, in a previous nationwide breast cancer audit, a HER2-outlier hospital critically evaluated their laboratory process and found that they used a different approach to HER2 positivity [49].

In conclusion, this nationwide study shows that there is limited absolute variation in ER-, PR- and HER2-receptor positivity rates between Dutch pathology laboratories in daily clinical practice. Yet, the considerable number of outlying laboratories shows that there is still room for improvement. Continuous monitoring and benchmarking of positivity rates may help to realize this and has been implemented in the Netherlands.
